# Multifunctional Reconfigurable Operations in an Ultra-Scaled Ferroelectric Negative Transconductance Transistor

**DOI:** 10.1021/acsnano.4c09598

**Published:** 2024-10-11

**Authors:** Zhongyunshen Zhu, Anton E. O. Persson, Lars-Erik Wernersson

**Affiliations:** Department of Electrical and Information Technology, Lund University, Lund 221 00, Sweden

**Keywords:** multifunctional, ferroelectric, negative transconductance, reconfigurable, data search, signal processing

## Abstract

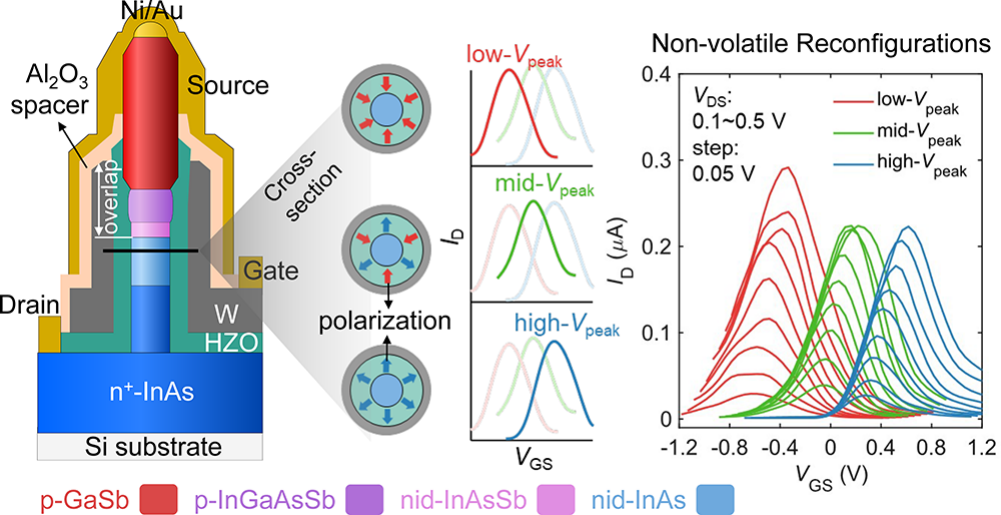

The integration of functional materials into electronic devices has become a key approach to extending Moore’s law by increasing the functional density of electronic circuits. Here, we present a device technology based on ultrascaled ferroelectric, antiambipolar transistors (ferro-AAT) with robust negative transconductance, enabling a wide range of reconfigurable functionalities with applications in both the digital and analog domains. The device relies on the integration of a hafnia-based ferroelectric gate stack on a vertical nanowire tunnel field-effect transistor. Through intentional gate/source overlap and tunnel-junction engineering, we demonstrate enhanced antiambipolarity with a high negative transconductance that is reconfigurable using the nonvolatile remanent polarization of the ferroelectric. Experimental validation highlights the versatility of this ferro-AAT in two implementation scenarios: content addressable memory (CAM) for high-density data search and reconfigurable signal processing in analog circuits. As a single-transistor cell for CAMs, the ferro-AAT shows subpicojoule operation for one search with a compact footprint of ∼0.01 μm^2^. For single-transistor-based signal modulation, multistate reconfigurations and high power conversion (>95%) are achieved in the ferro-AAT, resulting in a significant reduction in the complexity of analog circuit design. Our results reveal that the distinctive device properties allow ferro-AATs to operate beyond conventional transistors with multiple reconfigurable functionalities, ultrascaled footprint, and low power consumption.

## Introduction

As the conventional miniaturization of transistors—the building blocks of microelectronics—is nearing their physical scaling limit,^1,2^ multifunctional devices that operate beyond traditional transistors have become increasingly attractive for enhancing the functional density of computer chips.^3,4^ This advancement is essential to meet the growing demands of emerging information technologies driven by data-centric computation and storage such as artificial intelligence (AI) and Internet-of-Things (IoTs). Antiambipolar transistors (AATs) are one of the emerging device classes that have attracted tremendous interest due to their special transfer characteristics with high nonlinearity. Unlike conventional transistors in which the current increases continuously with voltage, an AAT exhibits a peak current at a certain gate voltage while the current decreases at voltages above and below this peak, resulting in a Λ-shape transfer curve as well as a negative transconductance (*g*_m_). This characteristic, originating from a gate-tunable p–n junction, enables a range of promising functionalities such as multivalued logic,^5−7^ analog signal modulation,^8−10^ and neuromorphic computing,^11,12^ leading to enhanced data processing capability while reducing device footprint.

To further extend functionality, reconfigurable AATs are appealing devices with potential for dynamic adaptability across diverse applications.^13^ Due to the superior electronic properties in the atomically thin layer, two-dimensional (2D) materials have recently been investigated for such emerging device technologies by introducing a dual-gate architecture.^14,15^ Despite demonstration of highly reconfigurable applications such as polymorphic logic gates^14^ and multifunctional image processing,^15^ most 2D-based AATs suffer from power-inefficient high supply and control voltages as well as volatile reconfigurability.^13^ Recent efforts to achieve nonvolatile reconfigurability in AATs through floating gates^16^ or charge trapping in the gate dielectric^15^ have shown promise. However, these methods require high-voltage or long-duration programming pulses, hindering their feasibility for practical applications. Therefore, the development of emerging device technologies and material integrations for reconfigurable AATs is crucial to achieve faster operation, improved energy efficiency, and nonvolatility of their multifunctionality.

In this work, we demonstrate multistate reconfigurable operation of a ferroelectric-gated AAT (ferro-AAT) by integrating a Zr-doped HfO_2_ (HZO) gate oxide onto a vertical gate-all-around nanowire tunnel FET (TFET). TFETs typically have a p-i-n structure, which resembles the gate-controlled p–n junction in AATs. By partially gating the source segment to create a gate/source (G/S) overlap structure, antiambipolar operation can be realized by modulating the band-to-band tunneling (BTBT) path.^17^ Here, we optimize the doping position in the source segment of the TFET to achieve robust antiambipolarity with both a high peak–valley current ratio (PVCR) and negative *g*_m_. The use of ferroelectric gate oxide allows the device to nonvolatilely reconfigure the threshold voltage (*V*_T_) with substantially reduced programming pulse width and amplitude. Depending on the ferroelectric polarization state, we realize three reconfigurable states using the same bias while retaining strong antiambipolarity extending our previous studies.^18^ Furthermore, we experimentally demonstrate that our ferro-AATs can be implemented for high-density content-addressable memory (CAM) and low-power reconfigurable analog signal processing. The results show that our ferro-AATs are able to extend the transistor functionality to diverse application scenarios requiring high energy and area efficiency such as memories and analog circuits.

## Results and Discussion

### PVCR Engineering by Tuning the Ferro-AAT Structure

**Figure 1:**
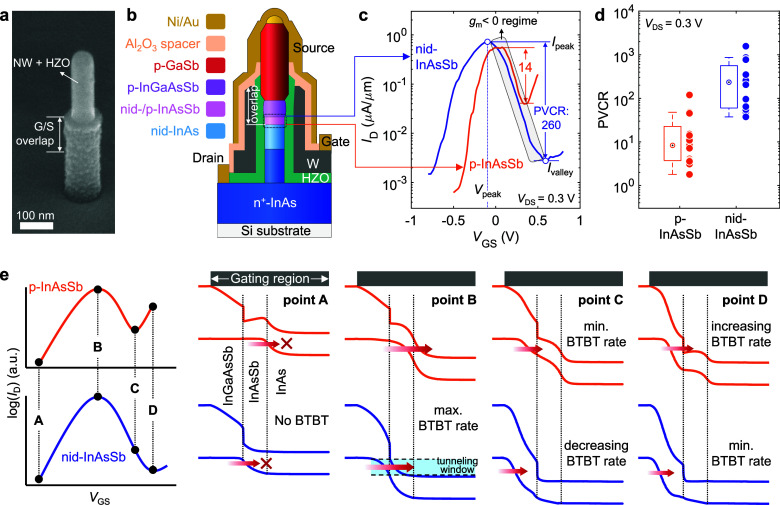
Device structure with G/S overlap and source doping effect. (a) Scanning electron microscopy (SEM) image of the vertical TFET nanowire post the fabrication process of gate-length definition (see Method) showing a clear G/S overlap. (b) Device schematic of a ferro-AAT with InAs/InAsSb/InGaAsSb/GaSb vertical nanowire and G/S overlap structure. Annotation “nid-/p-InAsSb” denotes two different samples with either nid-InAsSb or p-InAsSb segment in the nanowire structure. (c) As-fabricated (before ferroelectric switching) transfer characteristics of two ferro-AATs with a p-InAsSb or nid-InAsSb segment, respectively. (d) Statistics of PVCR in the two samples. (e) Band diagram of the p-InGaAsSb/p-(or nid-)InAsSb/nid-InAs heterojunction with BTBT transport mechanism at different *V*_GS_ bias points, which explains the cause of the improved PVCR in the ferro-AAT when using a nid-InAsSb segment. The higher transfer curve with p-InAsSb (orange line) in the schematic does not indicate a higher *I*_D_ but is used for better visualization.

Figure 1a shows a ferroelectric-gated nanowire TFET with evident G/S overlap after gate-length definition. Figure 1b illustrates a fabricated ferro-AAT (process flow in Figure S1) consisting of InAs/InAsSb/InGaAsSb/GaSb TFET nanowires. Such heterostructure TFETs with thin bilayer high-κ dielectric have demonstrated higher subthermionic tunneling current density compared to other reported TFETs.^19^ In this study, we grew the same nanowire heterostructure but replaced the dielectric with 13 nm-thick HZO ferroelectric and intentionally increased the gate length to partly overlap the source segment during fabrication. Furthermore, while keeping a similar G/S overlapped area, in a second device structure, the nanowire structure near the tunnel junction was slightly tuned by delaying the introduction of p-type dopant (Zn) in the source adjunct to the InAs channel. This delay allows the p-InAsSb to be replaced with a nonintentionally doped (nid) InAsSb segment (Figure 1b).

First, we characterize the ferro-AATs before any ferroelectric switching (as-fabricated). A more symmetric antiambipolar behavior is observed in the transfer characteristic in the device with a nid-InAsSb segment than with p-InAsSb (Figure 1c). Moreover, the existence of nid-InAsSb in the gated region suppresses the valley current (*I*_valley_) while retaining an almost identical peak current (*I*_peak_), leading to a significantly improved PVCR (defined by *I*_peak_/*I*_valley_) of 260 (Figure 1c). This value is in line with other reported AATs^5,6,12,20−22^ and typically higher than a two-terminal tunnel diode (e.g., Esaki diode) with a similar *I*_peak_ level in the negative-differential-resistance region at room temperature.^13^ The statistical results presented in Figure 1d show that such structure engineering in ferro-AATs leads to over 1 order of magnitude higher PVCR compared to that with p-type doped InAsSb. On the other hand, a large negative *g*_m_ and a symmetric transfer curve are also desired for nonlinear analog applications such as frequency multiplication.^10,23^ Our ferro-AATs with the nid-InAsSb segment demonstrate an exceptional PVCR of over 1000 and a maximal negative *g*_m_ exceeding 0.5 μS with a highly symmetric transfer curve (Figure S3). These results highlight a great balance between PVCR and negative *g*_m_ in contrast with other AATs utilizing various materials and device technologies, particularly considering the device footprint (Table S1).^5,6,12,20−22^

The band diagrams shown in Figure 1e elucidate the BTBT transport mechanism in ferro-AATs with p-InAsSb and nid-InAsSb segments. When *V*_GS_ is biased from point A to B, the tunneling window opens, resulting in an increased BTBT current in both cases. Due to the presence of a nid-InAsSb segment, the BTBT mainly occurs between p-InGaAsSb and nid-InAsSb, which differs from the case with p-InAsSb. When further increasing *V*_GS_ higher than *V*_peak_ (defined by *V*_GS_ at *I*_peak_, see Figure 1c) from point B to C, the band edges in the gated source move down to suppress the BTBT rate, leading to a reduction in *I*_D_. In the case of p-InAsSb, the BTBT rate reaches the minimum at point C and starts increasing again with *V*_GS_ due to the narrow band gap of InAsSb in the gated source, resulting in a higher *I*_valley_. However, in the case using nid-InAsSb, the gated source is mainly p-InGaAsSb with a wider band gap, thus decreasing the BTBT rate further and approaching the minimum at a higher *V*_GS_ (point D). Therefore, a lower *I*_valley_ is obtained, which leads to an increased PVCR. Moreover, further *I*_D_ increase is inhibited after the current valley (point D) due to the wide band gap in the gated p-InGaAsSb segment, leading to enhanced symmetry in the transfer curve (Figure 1c).

### Multistate Reconfigurable Antiambipolarity

**Figure 2:**
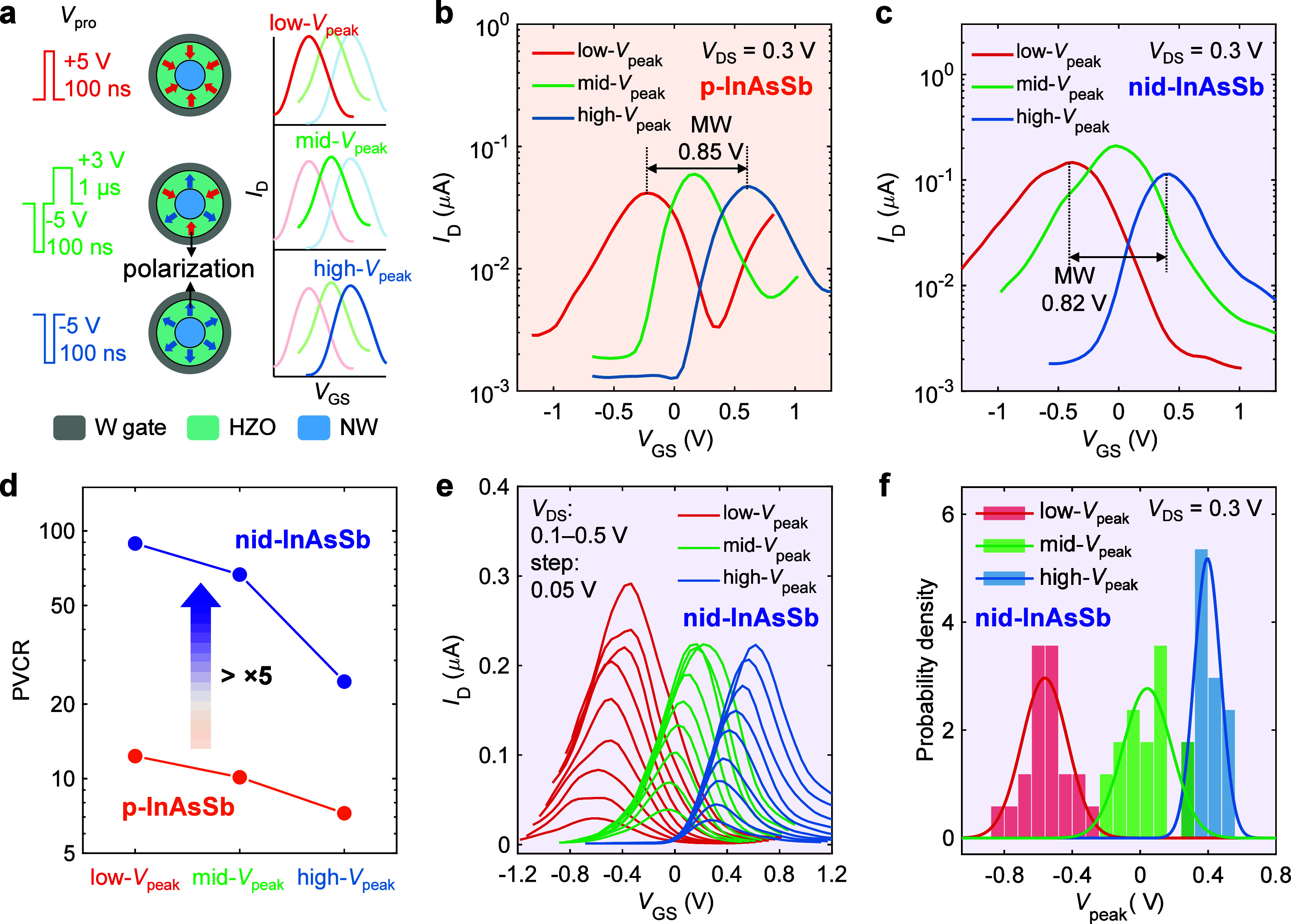
Nonvolatile reconfigurability in ferro-AATs. (a) Schematics of the polarization switching controlled by *V*_pro_ to the ferroelectric gate for the low-, mid-, and high-*V*_peak_ state. The source and drain terminals are grounded when *V*_pro_ is applied. Three-state reconfigurable transfer characteristics of a ferro-AAT from the sample with (b) p-InAsSb and (c) nid-InAsSb segment. (d) PVCR comparison between two samples in the three states. (e) Transfer characteristics at various *V*_DS_ of the device with nid-InAsSb, showing a symmetric bell shape of *I*_D_–*V*_GS_ in the on-state. (f) Statistical distribution of *V*_peak_ in the three states based on 20 devices from the sample with nid-InAsSb.

Next, we characterize the ferro-AATs with nonvolatile reconfigurability that is enabled by the ferroelectric HZO. The ferroelectricity in the HZO film was verified by polarization-voltage hysteresis and capacitance–voltage characteristics (Figure S2). By applying a programming voltage pulse (*V*_pro_) to the gate, the remanent polarization in the gate-all-around ferroelectric film can be switched, leading to a reconfigurable antiambipolar transfer characteristic in these particular devices with three states, i.e., low-, mid-, and high-*V*_peak_ state (Figure 2a). Although nanosecond or microsecond pulses are used here, the programming speed of HZO-based ferroelectric FETs has been experimentally verified to be as fast as subnanoseconds (300 ps).^24^ Furthermore, ferroelectric memories have shown the lowest write energy (1–10 fJ) among all other emerging nonvolatile memories, indicating a high energy efficiency.^25^ We first compare the transfer characteristics of two samples with different device structures (p-InAsSb and nid-InAsSb) after ferroelectric switching. In contrast to the device with p-InAsSb, the ferro-AAT with nid-InAsSb exhibits more symmetric transfer curves (Figure 2b,c) and substantially improved PVCR (Figure 2d) in all three states owing to the *I*_D_ suppression at high *V*_GS_, in good agreement with the results before ferroelectric switching. The reduced PVCR in both samples mainly results from the increased minimum current including *I*_valley_ after ferroelectric switching.^26,27^ Moreover, a similar memory window (MW) in both devices is observed (Figure 2b,c) as the ferroelectric gate stack is identical. These results indicate that the antiambipolarity highly depends on the heterostructure at the tunnel junction rather than the ferroelectric gate, which mainly provides the nonvolatile reconfigurability in the ferro-AATs. Despite asymmetric N-shape transfer characteristics with a low PVCR, the ferro-AATs with p-InAsSb can be still utilized for multivalued logic circuits in which the negative *g*_m_ regime is usually designed for the middle logic value.^6,20^ Nevertheless, in this work, we mainly focus on the ferro-AAT with nid-InAsSb due to the improved antiambipolarity with highly symmetric transfer curves, which is typically more desired in AATs.^13^ Thus, in the following description, “ferro-AATs” refers to the one with nid-InAsSb unless otherwise stated.

Within an individual ferro-AAT, it is noticeable that the *I*_D_–*V*_GS_ curve is more symmetric in the mid-*V*_peak_ state than in the other two states (Figure 2c). As a result, both positive and negative *g*_m_ are similar in the mid-*V*_peak_ state while a higher negative *g*_m_ and a slightly higher positive *g*_m_ are observed in the low-*V*_peak_ and high-*V*_peak_ state, respectively (Figure S4a). Furthermore, the antiambipolarity of the ferro-AAT is retained over a wide *V*_DS_ range for all three states (Figure 2e) and the corresponding *V*_peak_ shifts positively when increasing *V*_DS_ while retaining a nearly identical MW of ∼0.9 V between the low- and high-*V*_peak_ state (Figure S4b). This originates from the fact that more vacant states are available for tunneling at larger *V*_DS_, and thus a higher *V*_GS_ is required to reach the maximum BTBT rate. The statistical distribution of *V*_peak_ in Figure 2f shows that the high-*V*_peak_ state has less device-to-device variation than that in the other two states in which a similar *V*_peak_ spread is found. A slightly higher device-to-device variation in the mid-*V*_peak_ state might be due to a lower stability of partial polarization in the ferroelectric HZO. For the high- and low-*V*_peak_ states, the growth and process of the nanowires rather than the ferroelectric gate may dominate the device-to-device variation. Overall, after device structure engineering, our ferro-AATs exhibit robust antiambipolarity and multistate nonvolatile reconfigurability.

We then investigated the dynamics and reliability of ferroelectric switching in ferro-AATs. The result of ferroelectric switching property clearly shows three states with abrupt transitions (Figure S5) that can be understood as the single domain switching existing in ferroelectric-gated TFETs.^27^ Moreover, the device reliability, including retention time and endurance, is measured at room temperature (Figure S5). All the *V*_peak_ are stable for >3000 s and can be possibly extrapolated up to 10 years (Figure S5a,b). The endurance reaches 10^4^ switching cycles with a slightly reduced MW and is followed by hard breakdown (Figure S5c,d), which is expected to be avoided by inserting a thick spacer between the gate and drain to reduce the planar capacitor area (see discussion in Figure S6 caption). The three *V*_peak_ states remain distinct until 10^4^ switching cycles, at which point the *V*_peak_ value of the mid-*V*_peak_ state becomes higher than that of the high-*V*_peak_ state, disabling the multistate operation. Additionally, *V*_peak_ in all three states shifts negatively when cycled, leading to potential cycle-to-cycle variation. It has been reported that the endurance can be potentially improved by engineering the HZO structures and interfaces.^28,29^ Nevertheless, the presented ferro-AATs can still be utilized in some applications that do not require heavy rewriting tasks such as data search with CAMs^30^ and preprogrammed data processing like image classification.^31^ Therefore, we primarily introduce the above application schemes that can be effectively implemented with our ferro-AATs, given their reconfigurable antiambipolarity, high efficiency in terms of area and energy, and suboptimal endurance. Temperature stability is another factor that needs to be considered for nonvolatile reconfigurable devices. Thanks to the BTBT transport mechanism, TFETs typically have a thermally stable performance.^32^ Therefore, the impact of temperature on device performance may be mainly attributed to the ferroelectric HZO film, having the potential to achieve higher temperature stability as compared to conventional ferroelectric FETs based on a thermal injection transport mechanism.

### Ferro-AAT-Based CAM Cell for High-Density Data Search

CAM is a hardware architecture that can perform parallel search in massive data sets, thereby being attractive in data-centric applications such as machine learning^33^ and big-data processing.^34^ As compared to the conventional CAM cells based on static random-access memories (SRAMs), emerging nonvolatile memories such as resistive RAMs^35,36^ and ferroelectric FETs^30,37^ have demonstrated higher scalability and improved energy efficiency; however, a single cell still requires at least two devices in those implementations.

**Figure 3:**
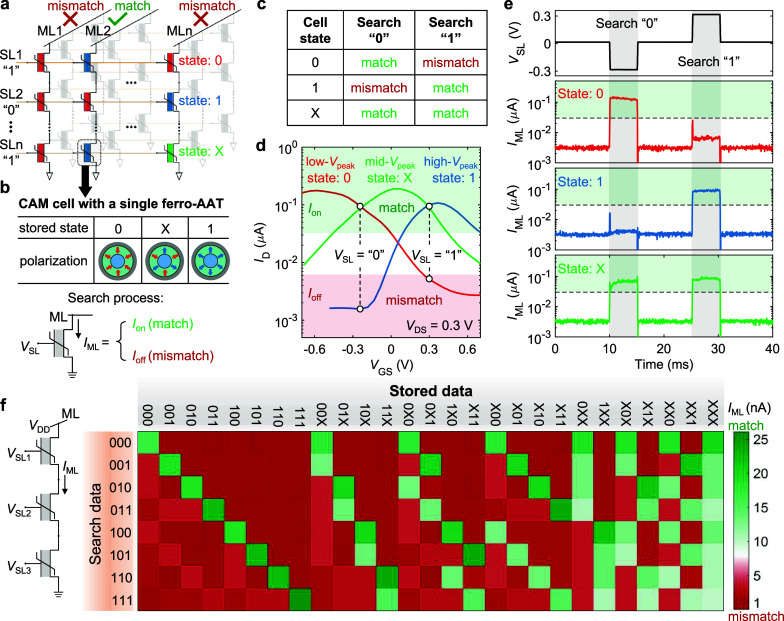
Implementation of NAND-type CAM cell with a single ferro-AAT. (a) Schematic of a high-density NAND-based CAM array with an n-bit search process at the SLs and n MLs using ferro-AATs. When all the ferro-AAT cells match the input at SLs, *I*_on_ will be sensed at the corresponding ML (ML2 in the schematic). (b) Three polarization states can be stored as state “0″, “X”, and “1″, referring to low-, mid-, and high-*V*_peak_ state, respectively. When the searching data matches the stored one, *I*_on_ is sensed for *I*_SL_ while *I*_off_ is obtained with mismatching. (c) Corresponding operation table of the ferro-AAT-based CAM cell. (d) Measured transfer characteristic in the three stored states with *V*_SL_ = “0” or “1” for searching “0” or “1”. Here, *I*_on_ is always sensed in the “X” state. (e) Waveforms of input *V*_SL_ and measured *I*_SL_ in the searching process for 1-bit ferro-AAT cell. Supply voltage *V*_DD_ = 0.3 V is only used during search. (f) 2D mapping result of 3-bit data search based on a simplified circuit configuration with three ferro-AATs in series. The currents are modeled using the load line analysis based on measured device with *V*_DD_ = 0.3 V and *V*_SL_ = ± 0.3 V representing 1 and 0, respectively.

To further scale down the cell size, we here utilize a single ferro-AAT as one cell for NAND-type CAM.^38^ In this NAND-type CAM architecture, a search query is the input to the search lines (SLs) in parallel to compare with the stored states in ferro-AAT cells in the CAM array, and the results are sensed by the current (*I*_ML_) through each match line (ML) consisting of the cells in series (Figure 3a). When all SL bits match the stored state in the ML, a high *I*_ML_ value will be obtained. The table in Figure 3b presents the three polarization states in a single ferro-AAT cell, corresponding to the stored state “0″, “1″, and “X” (do not care). In the “X” state, the search function always outputs a match regardless of the actual input data at the SLs. This property allows the “X” state to cover a broad memory range, which is useful for entries requiring to match several addresses or multiple patterns, thereby reducing the complexity of CAM.^39^ For a single cell, *I*_ML_ = *I*_on_ when the input bias at the SL (*V*_SL_) matches the stored value or “X”, and otherwise *I*_ML_ = *I*_off_. Figure 3c lists all of the search results based on three states for a single cell. The expected operation is achieved in one ferro-AAT by considering the low-, mid-, and high-*V*_peak_ state as the “0″, “X”, and “1” state, respectively (Figure 3d). To achieve comparable current levels in the low-*V*_peak_ and the high-*V*_peak_ state, a high PVCR is particularly desirable in the low-*V*_peak_ state, where the negative *g*_m_ region dominates. In this case, statistical analysis in the low-*V*_peak_ state reveals that the ferro-AAT sample with nid-InAsSb has a PVCR over 10 times higher and a slightly enhanced peak negative *g*_m_ compared to the one with p-InAsSb (Figure S7), indicating a higher feasibility for data search operation in the proposed CAM application.

Next, we implement the 1-bit data search process in the ferro-AAT with transient *I–V* characterization as shown in Figure 3e. The results reveal similar current levels of both *I*_on_ and *I*_off_ in all three reconfigurable states despite a slightly higher *I*_off_ in the “0” state. Due to the symmetric antiambipolar transfer characteristic in the mid-*V*_peak_ state as shown in Figure 3d, almost identical current levels are obtained when searching for either “0” or “1” (Figure 3e). Notably, several current spikes in the *I*_ML_ waveform are observed when *V*_SL_ switches (Figure 3e), which mainly originate from the large planar capacitance at the drain terminal. The search time can be potentially decreased below 1 ms as the rising time from off- to on-state is ∼100 μs. By using the *I–V* characteristics of the same measured device and the load line analysis (Note S1), the expected *I*_ML_ mapping result of 3-bit data search with all 27 data-stored configurations is successfully achieved based on the circuit configuration where three ferro-AATs are in series (Figure 3f). Despite the current variation in the three states (Figure S11), a distinct separation between the lowest matched *I*_ML_ (10 nA) and the highest mismatched *I*_ML_ (3.5 nA) is found (Figure 3f). The reduction of the maximum matched *I*_ML_ (*I*_on_) in Figure 3f is due to the reduced *V*_DS_ for each ferro-AAT as the supply voltage *V*_DD_ is fixed at 0.3 V, leading to a reduced power consumption of ∼6 nW.

Benefiting from the small footprint (∼0.01 μm^2^) of such single vertical nanowire architecture, the cell array for multibit CAM can be scaled up significantly with the same area, thereby being promising for a high-volume data search process. Furthermore, the NAND-type CAM architecture allows single cells to vertically stack in one ML, resulting in high-density 3D integration.^37^ This approach is particularly suitable for vertical gate-all-around nanowire transistors, which can achieve a footprint of 4*F*^2^, the theoretical minimum based on the smallest feature size of lithography (*F*). By using bottom-up epitaxy^40^ or top-down etching processes,^41^ multiple heterostructure ferro-AATs can be vertically stacked, enabling ultrahigh-density CAM arrays where a single nanowire with multibit SLs represents one entry address. In addition, the proposed NAND-type CAM based on ferro-AATs eliminates the precharge phase typically required in the NOR-type CAM, thus further improving energy efficiency.^42^ Such precharge-free property leads to an estimated search energy of ∼0.6 pJ with our ferro-AAT cells for a 3-bit query, in line with reported ferroelectric-based CAM cells.^43^ These two merits yield a higher area and energy efficiency as compared to SRAM-based CAM which typically requires multiple transistors per cell.^44^ Although the current device may be limited by its endurance due to the lack of a first spacer (Figure S6) as discussed previously, it might be still sufficient for data search as write operation in CAMs may not be as frequent as other memory applications.^30^

### Multimode Reconfigurable Signal Processing

**Figure 4:**
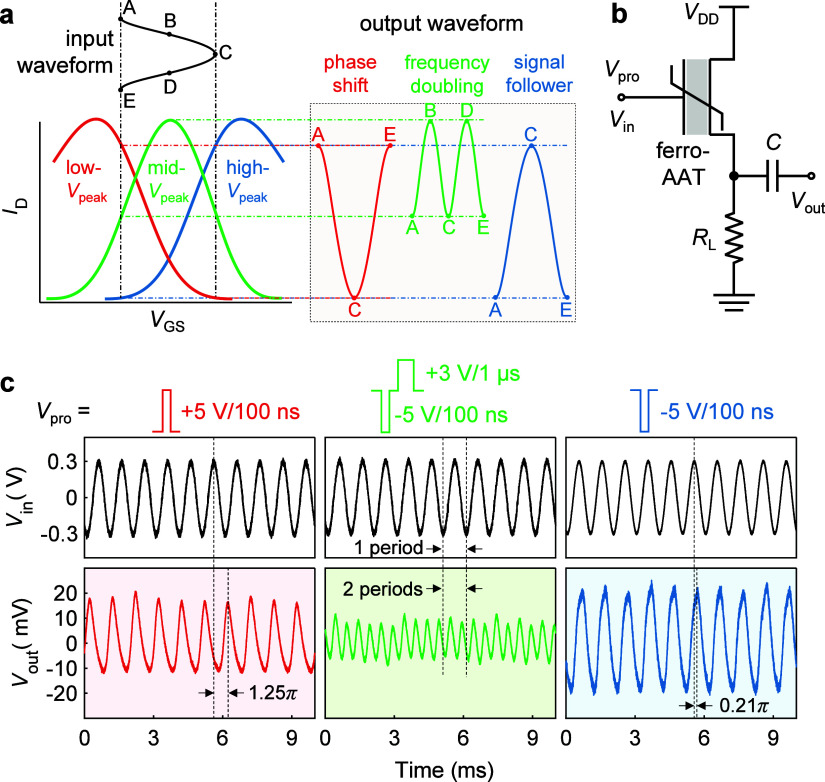
Multimode reconfigurable signal modulation with a single ferro-AAT. (a) Working principle of three signal modulations: phase shift, frequency doubling, and signal follower. (b) Measurement schematic of transient *V*_in_–*V*_out_ waveforms. Here, the load resistance (*R*_L_) is 8 MΩ and the capacitance (*C*) filters the DC component of the output signal. (c) Excerpted measured *V*_in_–*V*_out_ waveforms in three different modulation modes as illustrated in (a).

Another potential application scheme we demonstrate here with ferro-AATs is multimode reconfigurable analog signal modulation. Due to the symmetric transfer curve in AATs, single-transistor frequency doubling has been widely investigated.^9,10,18,45,46^ Recently, two-mode reconfigurable signal modulation using ferroelectric transistors has been reported with low programming power owing to their nonvolatility.^18,23,47^ To further add functionality, a three-mode reconfigurable transistor with a back gate for signal modulation has been demonstrated.^48^ However, a high back-gate bias is always required to program the modulation mode and must be maintained throughout the entire signal modulation process. Here, we realize the three-mode modulation functionality with nonvolatile reconfigurations in a single ferro-AAT. The working principle is elucidated in Figure 4a. An input analog signal (*V*_in_) is designed to oscillate near the current peak in the mid-*V*_peak_ state, meaning that a DC offset of *V*_peak_ in the mid-*V*_peak_ state needs to be applied. As shown in Figure 2f, such *V*_peak_ is close to zero, leading to a negligible DC offset in *V*_in_, which simplifies the biasing condition. Depending on the polarization state, three reconfigurable modulation modes can be realized. In the mid-*V*_peak_ state, the semicycle A–B-C of the *V*_in_ waveform will become a full cycle in the output waveform, resulting in frequency doubling. In the other two states, the *V*_in_ waveform operates in the linear regime of the transfer curves with either a negative-*g*_m_ (low-*V*_peak_ state) or positive-*g*_m_ (high-*V*_peak_ state) characteristic, leading to either a π phase shift or a signal follower mode at the output, respectively.

We implement reconfigurable analog signal modulation with a single ferro-AAT under the circuit configuration shown in Figure 4b, using a fixed *V*_in_ without an additional DC offset. The expected *V*_in_–*V*_out_ waveforms in the three modulation modes are shown in Figure 4c with the corresponding states whose transfer characteristics are presented in Figure S8a. To achieve appropriate *V*_out_ waveforms, the peak-to-peak voltage (*V*_PP_) of *V*_in_ should be within *V*_peak_ in the low- and high*-V*_peak_ state (gray region in Figure S8a). Thus, a *V*_PP_ of 0.6 V is selected for *V*_in_ to prevent distortion and suppression of harmonics. The amplitude of *V*_out_ in the high-*V*_peak_ state is slightly larger than that in the low-*V*_peak_ state (Figure 4c) due to a higher *g*_m_ in the n-type branch in the high-*V*_peak_ state (Figure S8b). Notably, a phase delay of 0.21π is observed in the high-*V*_peak_ state (signal follower), which mainly originates from the capacitive load at the output.^48^ This is verified in the low-*V*_peak_ state (phase shift) where a phase delay of 1.25π exists, leading to approximately a π phase shift between two states. The corresponding power spectra of the three modulation modes in which over 95% of output power is concentrated on the target output frequency indicate high spectral purity without the need for additional filters (Figure S9). This is particularly promising for the frequency doubling mode as unwanted harmonics are typically generated and require additional circuitry for filtering.^23^ In the operational range from −0.3 to 0.3 V, a high linearity of *g*_m_ as a function of *V*_GS_ is achieved in the mid-*V*_peak_ state (Figure S8b), leading to a nearly ideal parabolic transfer curve which is suggested for pure second harmonic generation.^10,23^

Compared to other single-transistor-based signal modulation devices,^9,10,23,45,46,48−50^ our multimode ferro-AATs exhibit outstanding performance in terms of low-power operation (nW range), high functional density, high spectral purity, and nonvolatile reconfigurability. Furthermore, such multifunctional analog signal modulators may enable analog in-memory computing in the frequency domain, leading to high energy and area efficiency for edge AI applications.^51^ Although the operating frequency in the presented ferro-AAT is limited to the kHz range due to large parasitic capacitance, further processing optimization such as inserting a low-κ spacer between electrode pads to suppress the parasitic capacitance can significantly raise the cutoff frequency up to the GHz range.^52^

## Conclusions

We report a ferro-AAT with multistate nonvolatile reconfigurability based on a heterostructure TFET. The device exhibits a symmetric antiambipolar transfer characteristic, featuring a high PVCR and a robust negative *g*_m_. The ferroelectric nature of the gate oxide enables the high-speed and low-power programming of three distinct polarization states. The vertical nanowire architecture minimizes the device footprint, facilitating a high-density integration for large-scale data processing applications. Given its potential in energy and area efficiency, we experimentally demonstrate that a single ferro-AAT can be implemented as an ultrascaled cell for NAND-type CAM and as a versatile analog device for reconfigurable signal processing. Our results highlight the potential of multistate ferro-AATs for implementing reconfigurable functionality in both digital and analog applications.

## Method

### Device Fabrication

The details of the key fabrication steps are listed in Figure S1. The fabrication started with the nanowire growth by metal–organic vapor-phase epitaxy (MOVPE) via a vapor–liquid–solid (VLS) process using Au particles. Prior to the nanowire growth, 24 nm-wide Au discs were prepatterned by electron-beam lithography on a 260 nm highly doped InAs buffer layer on a highly resistive Si substrate. A Sn-doped (n-type) InAs stem was grown for the drain at 470 °C. Next, nid-InAs followed by nid-InAsSb or Zn-doped (p-type) InAsSb was grown at the same temperature for the channel. Finally, Zn-doped (p-type) InGaAsSb and Zn-doped (p-type) GaSb segments were grown while heating up to and at 515 °C for the source. After the nanowire epitaxy, 3 cycles of digital etch with sequential repetition of ozone exposure and wet etch in citric acid were employed to reduce the nanowire channel diameter to ∼23 nm as well as oxide removal at the channel interface to improve the electrostatics.^53^ As the gate oxide, a 13 nm-thick (confirmed by ellipsometer) HZO was deposited at 200 °C using thermal atomic layer deposition. A 60 nm-thick W gate-metal was then sputtered and defined to overlap the source segment in the nanowire using a UV-lithography S1813 (photoresist) mask and back-etch in a reactive ion etch (RIE) system with plasma SF_6_:Ar. After both gate pad definitions and via the opening process, the top HZO was wet etched by HF 1:400 to expose the source contact region using the S1813 mask. The sample was then annealed in a N_2_ ambient at 450 °C for 30 s using rapid thermal annealing (RTA) to crystallize HZO film with a partial orthorhombic phase^54^ that has been believed to mainly contribute ferroelectricity. The sample was finalized by contact metallization with 10 nm/200 nm Ni/Au bilayer metal, and 10 nm Al_2_O_3_ was utilized as the top spacer to isolate the gate.

### Electrical Characterizations

An MPI TS2000-SE probe station along with a Keysight B1500A parameter analyzer was employed for electrical characterizations. A B1530A waveform generator module (WGFMU) was used for the pulsed *I–V* measurements (*I*_D_–*V*_GS_ transfer characteristics) as well as for fast voltage pulses (*V*_pro_) to set the remanent ferroelectric polarization in the HZO gate. The sinusoidal waveforms of *V*_in_(*t*) and *V*_out_(*t*) were generated and sensed by a RIGOL DG1022Z Function Generator and a Rohde & Schwarz RTO Digital Oscilloscope, respectively.

## Supporting Information Available

Benchmarking the presented AATs against other reported ones; detailed process flow for vertical nanowire ferro-AAT fabrication; modeling methodology of 3-bit CAM system; extra material and electrical characterization supporting the conclusions (PDF)
