# Supplementary material for: Multifunctional Reconfigurable Operations in an Ultra-Scaled Ferroelectric Negative Transconductance Transistor
Source: ACS Nano. 2024 Oct 11;18(42):28977–85. doi: 10.1021/acsnano.4c09598 (PMC11503915; doi:10.1021/acsnano.4c09598)
Supplement: Supplementary file 1 — nn4c09598_si_001.pdf [file nn4c09598_si_001.pdf]

Supporting Information

Multifunctional Reconfigurable Operations in an Ultra-Scaled Ferroelectric Negative Transconductance Transistor

Zhongyunshen Zhu, Anton E. O. Persson⊥, and Lars-Erik Wernersson*

Department of Electrical and Information Technology, Lund University, 221 00 Lund, Sweden

* Email: zhongyunshen.zhu@eit.lth.se

⊥ Current address: Department of Electrical Engineering, Stanford University. Stanford, California 94305, United States of America

Content:

Table S1

Figures S1-S11

Note S1

References 1-12

Table S1. Comparison of recently reported AATs with various device architectures.

Device structure	Material	V_{DS} (V)	I_{peak} (nA)	PVCR	$-g_m$ (μS)	Device footprint [3)]	Ref.
ultra-thin body MOSFET	Si	0.3	0.2	$> 10^4$	5.4×10^{-3}	8.4 μm^2	[1]
2D/2D heterostructures	$MoS_2/MoTe_2$	1	5	10^3	6×10^{-4}	~25 μm^2	[2]
	p-BP/n-MoS_2	1	200	~100	0.4	~2 μm^2	[3]
1D/2D heterostructures	SWCNTs [1)]/MoS_2	1	1000	10~100 [2)]	1.25	~1000 μm^2	[4]
Organic FET	DNTT/PTCDI-C13	50	600	~100	0.15	~1.5×10^5 μm^2	[5]
	α-6T/PTCDI-C8	10	20	N.A.	0.04	5.4×10^4 μm^2	[6]
**III-V nanowire heterostructure**	**InAs/InAsSb/ InGaAsSb/GaSb**	**0.3**	**50**	**260**	**0.2**	**~0.01 μm^2**	**This work [4)]**

[1)] Single-walled carbon nanotube (SWCNT).

[2)] Varying with a back gate.

[3)] For ungiven numbers in the text, device area is determined by the given microscope image.

[4)] Average values before ferroelectric switching are displayed. Maximum values of PVCR (> 1000) and negative g_m (> 0.5 μS) are shown in Figure S2.

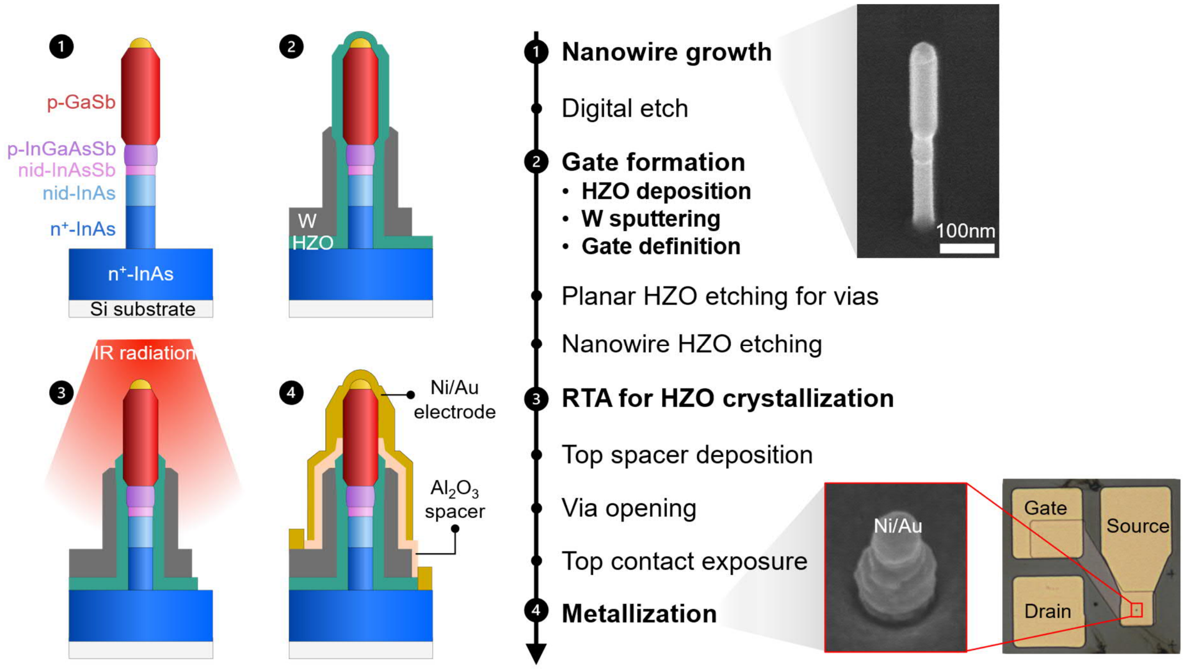

Figure S1. Detailed process flow of ferro-AATs on Si. SEM images including III-V TFET nanowires post MOVPE growth and the final ferro-AAT device are shown. The single nanowire device layout is displayed by optical microscopy.

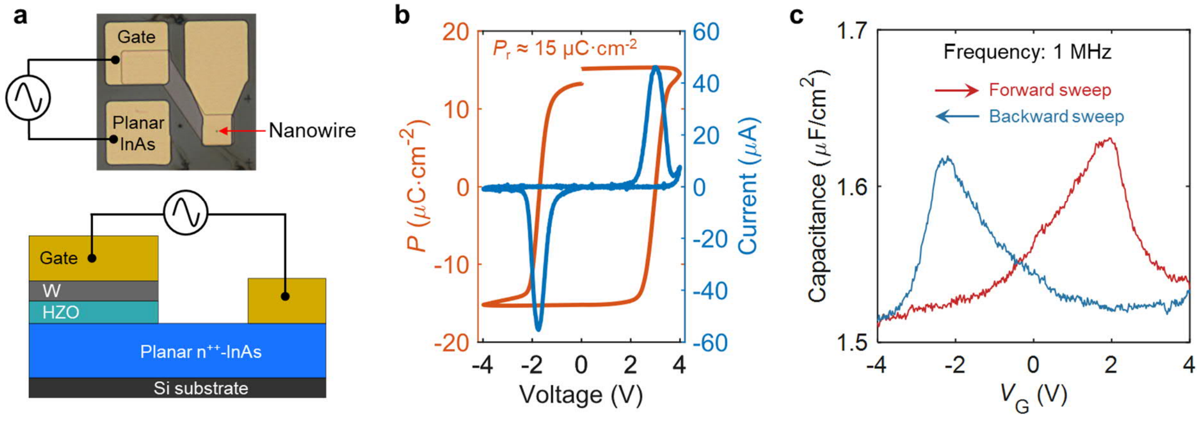

Figure S2. Material properties of ferroelectric HZO on InAs on the ferro-AAT sample. (a) Schematic of the positive-up-negative-down (PUND) and *C*-*V* measurements on a planar ferroelectric MOSCAP on the same ferro-AAT sample. (b) *P*-*V* hysteresis based on PUND measurement. The asymmetry in coercive field may result from the asymmetric W/HZO/InAs capacitor structure. (c) *C*-*V* characteristic with butterfly-shaped curves at 1 MHz, indicating the presence of ferroelectricity in the HZO film. Previous work has shown detailed structure characterizations including X-ray diffraction (XRD) and transmission electron microscopy (TEM) on an identical MOSCAP structure with identical deposition conditions[7].

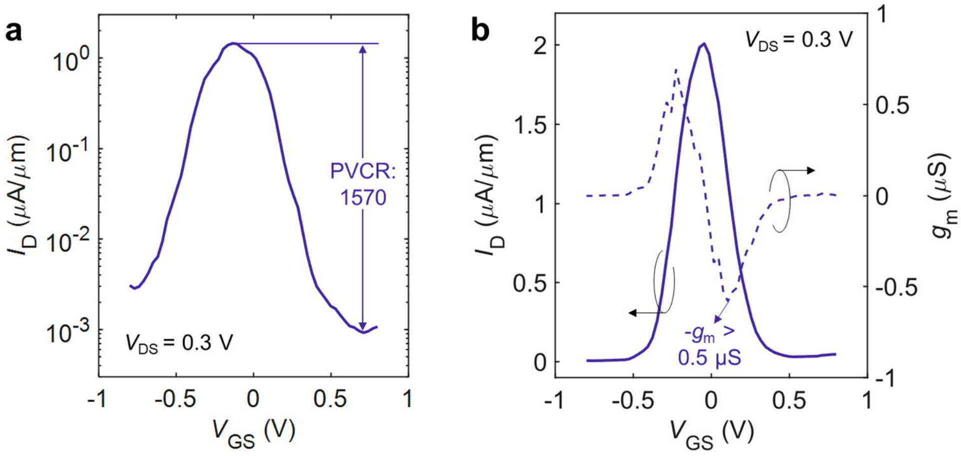

Figure S3. Extra electrical results in ferro-AATs before ferroelectric switching. (a) The highest PVCR >1000 is achieved in pristine (as-fabricated) ferro-AAT with a nid-InAsSb segment. (b) Another ferro-AAT on the same sample shows a negative g_m greater than 0.5 μS.

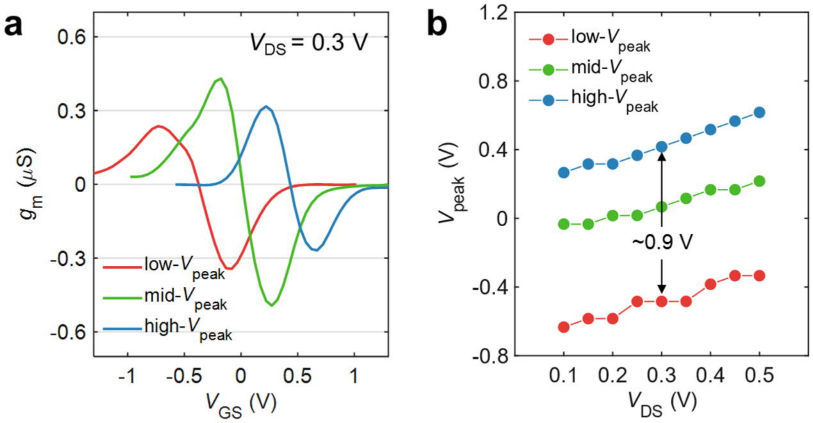

Figure S4. Reconfigurable properties in the ferro-AAT with nid-InAsSb segment. (a) g_m as a function of V_{GS}. (b) V_{peak} as a function of V_{DS} in the three states extracted from Figure 2e.

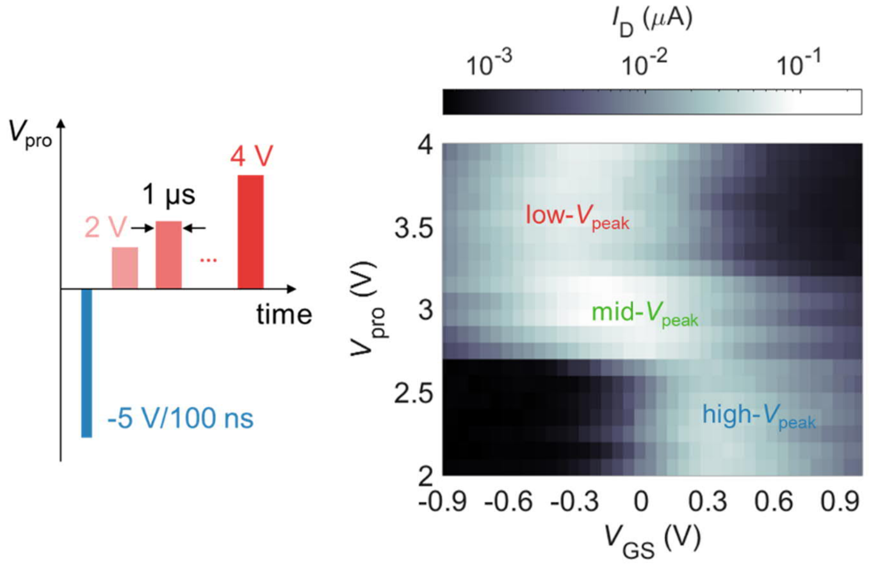

Figure S5. The ferroelectric switching behavior with progressively increased V_{pro} pulses varying from 2 V to 4 V with pulse width of 1 μs after a negative reset pulse of -5 V/100 ns. After each program pulse, the transfer characteristic is read by executing I_D-V_{GS} sweep, resulting in an I_D map with respect to V_{pro} and V_{GS}. The result shows a clear V_{peak} transition from high-V_{peak} state to low-V_{peak} state with a programming window of ~0.5 V for intermediate states including the mid-V_{peak} state.

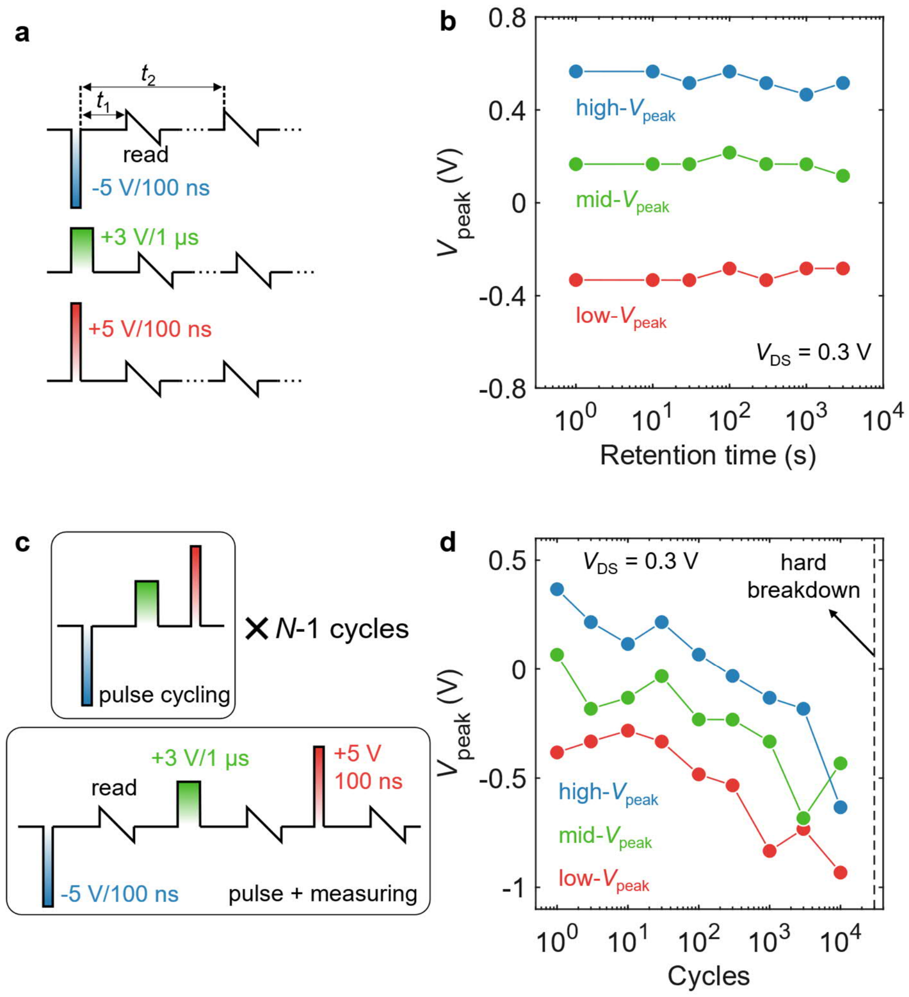

Figure S6. Device reliability. (a) Pulse scheme used for retention measurement. In this case, t_1 = 1 s, t_2 = 10 s, and so forth. (b) V_{peak} extracted from each transfer characteristic read after certain retention time. V_{peak} remains almost identical for each state for the measured retention time of 3000 s. Such retention stability in ferro-AATs with similar device structure has been shown up to 20 days with possible extrapolation to 10 years[8]. (c) Pulse scheme used for the endurance measurement. *N* denotes the cycle number. First, *N*-1 pulse cycles without reading scheme are carried out. After that, a one-write-one-read measurement scheme for each state is employed to determine V_{peak}. Such a non-destructive read operation does not affect the writing process as the voltage range is well below the coercive voltages of the HZO film. (d) The corresponding results

of V_{peak} as a function of cycles. Notably, the V_{peak} shifts negatively in the three states, which is mainly due to a negative V_T shift originating from additional charge trapping/de-trapping during cycling, leading to an increase in cycle-to-cycle variation for single devices. Despite this, a distinct memory window still exists up to 10^4 cycles. However, V_{peak} in the mid-V_{peak} state tends to be higher than that in the high-V_{peak} state after 10^4 cycles, which complicates the multi-state functionality. Interface engineering may be required to retain the V_{peak} position and thus further improve the endurance[9,10]. The measured device has a hard breakdown after 3×10^4 cycles due to the leakage from the large planar metal-ferroelectric-semiconductor (MFS) capacitor between the gate and the drain, which is much larger than that in the nanowire gate region. Since the endurance to hard breakdown is significantly area dependent[11], insertion of a spacer between the gate and drain metals may prevent breakdown before the memory window is gone. Also, a low-*k* spacer is helpful to substantially reduce the parasitic capacitance originating from the high-permittivity gate oxide between the electrode pads, thus increasing the operation frequency of the device[12].

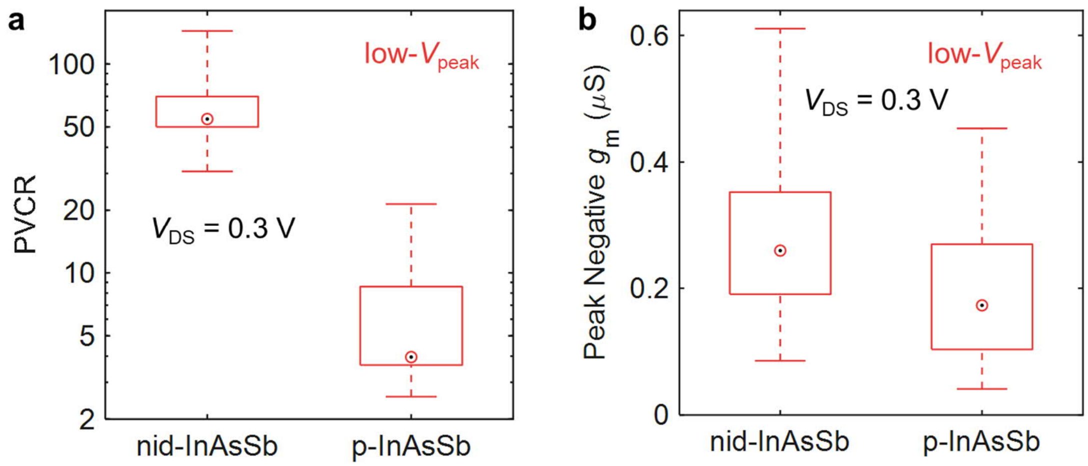

Figure S7. Comparison of two samples in the low-V_{peak} state in terms of (a) PVCR and (b) peak negative g_m based on statistics. While the negative peak g_m increases only slightly in the sample with nid-InAsSb compared to that in the sample with p-InAsSb, the PVCR improves more than one order of magnitude in the low-V_{peak} state. This is mainly a result from I_{valley} suppression in the sample with nid-InAsSb. Such high PVCR and negative g_m in the low-V_{peak} state leads to feasible implementations of ferro-AATs for

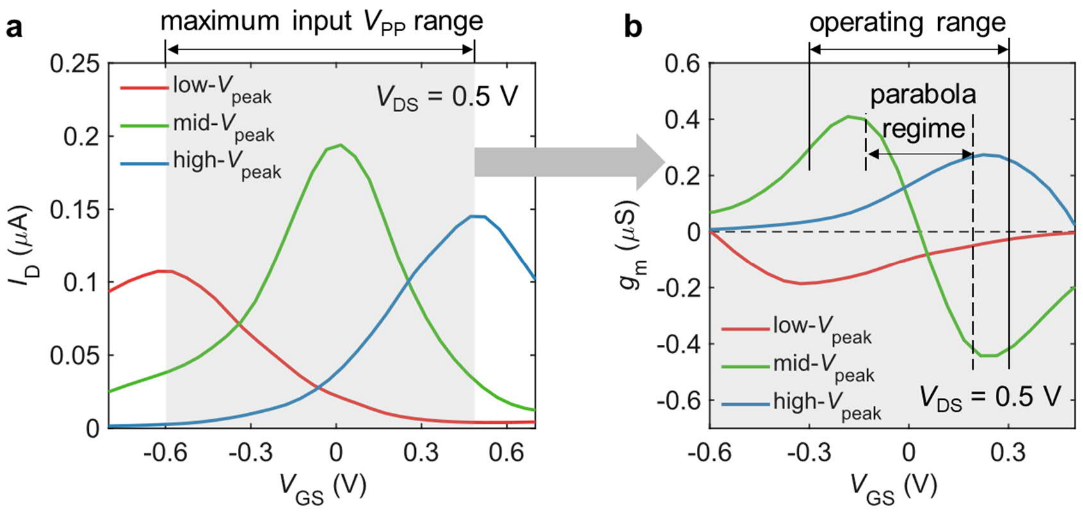

Figure S8. Transfer characteristics of three reconfigurable states for different signal processing in presented Fig. 4 of the main manuscript. (a) I_D-V_G transfer characteristics. To avoid large distortion and suppress the harmonics of V_{out}, the peak-to-peak voltage (V_{PP}) of the input signal should be within two V_{peak} as indicated in the grey region. (b) g_m-V_G transfer characteristics of the grey region shown in (a) where negative and positive g_m are obtained in the low- and high-V_{peak} state, respectively, which corresponds to phase shift and signal follower. In the case of the mid-V_{peak} state, frequency doubling can be realized. To suppress unwanted harmonics, a parabolic I_D-V_{GS} curve is ideally desired to only generate the doubled frequency from the input signal[13]. In the mid-V_{peak} as shown in (b), the linear dependency of g_m-V_G indicates the parabola region (-0.1~0.2 V) in which the input frequency can be in principle doubled in the output signal without generation of other harmonics. In our operating range of V_{GS} = [-0.3, 0.3] V, a near ideal parabolic transfer curve is still obtained due to the high linearity of g_m.

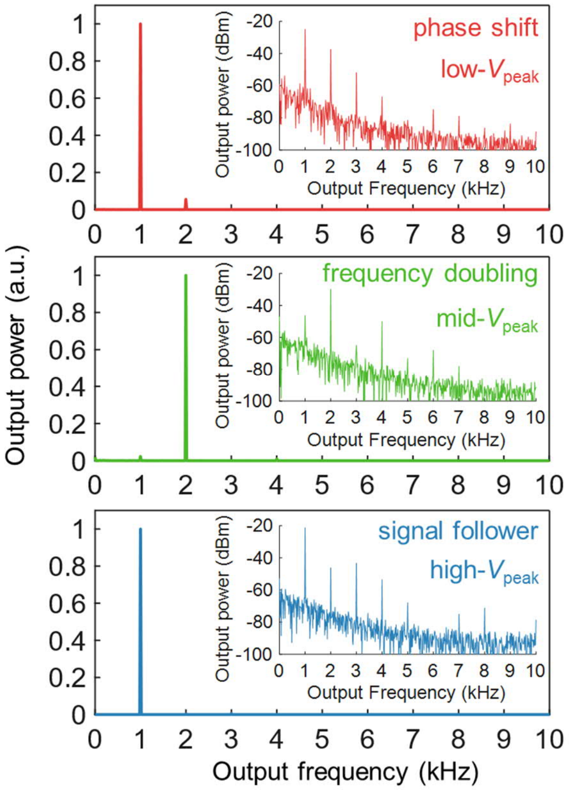

Figure S9. Power spectra of the output signal in the three reconfigurable states. The frequency-domain spectra are obtained by using FFT algorithm based on transient V_{in}-V_{out} measurements from the oscilloscope. The result indicates an excellent suppression of undesired harmonics with more than 95% output power concentrated at the desirable frequency in all three modes.

Note S1. Modeling of a 3-bit NAND-type CAM based on the measured ferro-AAT

We mainly use load line analysis to model 3-bit NAND-type CAM based on a circuit with three ferro-AATs in series. Here, we exclude the selector transistors at the drain/source side and peripheral circuits such as sensing amplifiers to simplify the model (see Figure S10).

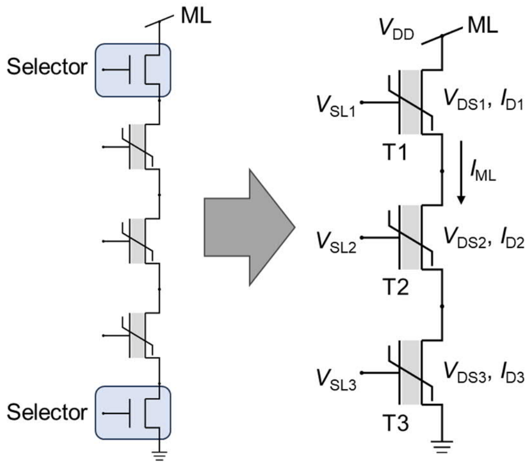

Figure S10. Schematic of the circuit configuration based on ferro-AATs for a 3-bit NAND-type CAM.

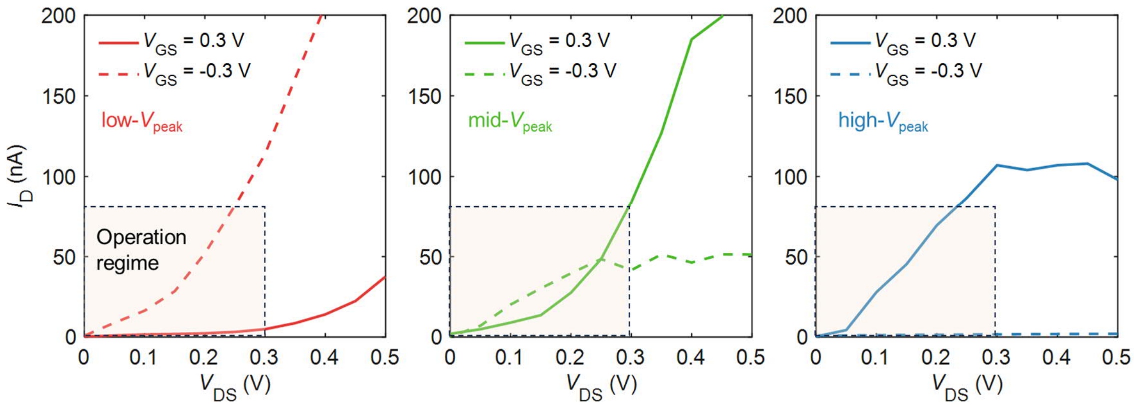

Figure S11. Output characteristics of the ferro-AAT in the three states at V_{GS} = ±0.3 V. The rectangles represent the operation regime corresponding to V_{DD} = 0.3 V.

In the above circuit configuration, we can obtain the following conditions:

1) $V_{DD} = V_{DS1} + V_{DS2} + V_{DS3}$

2) $I_{ML} = I_{D1} = I_{D2} = I_{D3}$.

Furthermore, the output characteristics in the three reconfigurable states demonstrate the relationship between I_D and V_{DS}. Figure S11 shows the I_D-V_{DS} characteristics measured at V_{GS} = ±0.3 V (V_{SL}) in each state. Combined with the aforementioned conditions, I_{ML} can be obtained with any possible search line inputs (V_{SL}) in any reconfigurable states. Notably, too large V_{DS} may degrade the performance in the mid-V_{peak} state due to asymmetry of the transfer curves when increasing V_{DS} (see Figure 3a), which mainly originates from an increased I_{valley} with V_{DS}. Therefore, our ferro-AATs are more suitable for low-power CAM operations.

References

(1) Lee, S.; Lee, Y.; Kim, C., Extraordinary Transport Characteristics and Multivalue Logic Functions in a Silicon-Based Negative-Differential Transconductance Device. *Scientific Reports* **2017,** *7* (1), 11065.

(2) Duong, N. T.; Lee, J.; Bang, S.; Park, C.; Lim, S. C.; Jeong, M. S., Modulating the Functions of MoS2/MoTe2 van der Waals Heterostructure via Thickness Variation. *ACS Nano* **2019,** *13* (4), 4478-4485.

(3) Huang, M.; Li, S.; Zhang, Z.; Xiong, X.; Li, X.; Wu, Y., Multifunctional high-performance van der Waals heterostructures. *Nature Nanotechnology* **2017,** *12* (12), 1148-1154.

(4) Beck, M. E.; Shylendra, A.; Sangwan, V. K.; Guo, S.; Gaviria Rojas, W. A.; Yoo, H.; Bergeron, H.; Su, K.; Trivedi, A. R.; Hersam, M. C., Spiking neurons from tunable Gaussian heterojunction transistors. *Nature Communications* **2020,** *11* (1), 1565.

(5) Yoo, H.; On, S.; Lee, S. B.; Cho, K.; Kim, J.-J., Negative Transconductance Heterojunction Organic Transistors and their Application to Full-Swing Ternary Circuits. *Advanced Materials* **2019,** *31* (29), 1808265.

(6) Hayakawa, R.; Honma, K.; Nakaharai, S.; Kanai, K.; Wakayama, Y., Electrically Reconfigurable Organic Logic Gates: A Promising Perspective on a Dual-Gate Antiambipolar Transistor. *Advanced Materials* **2022,** *34* (15), 2109491.

(7) Persson, A. E. O.; Athle, R.; Littow, P.; Persson, K.-M.; Svensson, J.; Borg, M.; Wernersson, L.-E., Reduced annealing temperature for ferroelectric HZO on InAs with enhanced polarization. *Applied Physics Letters* **2020,** *116* (6).

(8) Zhu, Z.; Persson, A. E. O.; Wernersson, L.-E., Reconfigurable signal modulation in a ferroelectric tunnel field-effect transistor. *Nature Communications* **2023,** *14* (1), 2530.

(9) Chan, C. Y.; Chen, K. Y.; Peng, H. K.; Wu, Y. H. In *FeFET Memory Featuring Large Memory Window and Robust Endurance of Long-Pulse Cycling by Interface Engineering using High-k AlON*, 2020 IEEE Symposium on VLSI Technology, 16-19 June 2020; 2020; pp 1-2.

(10) Ni, K.; Sharma, P.; Zhang, J.; Jerry, M.; Smith, J. A.; Tapily, K.; Clark, R.; Mahapatra, S.; Datta, S., Critical Role of Interlayer in Hf0.5Zr0.5O2 Ferroelectric FET Nonvolatile Memory Performance. *IEEE Transactions on Electron Devices* **2018,** *65* (6), 2461-2469.

(11) Francois, T.; Grenouillet, L.; Coignus, J.; Vaxelaire, N.; Carabasse, C.; Aussenac, F.; Chevalliez, S.; Slesazeck, S.; Richter, C.; Chiquet, P.; Bocquet, M.; Schroeder, U.; Mikolajick, T.; Gaillard, F.; Nowak, E., Impact of area scaling on the ferroelectric properties of back-end of line compatible Hf0.5Zr0.5O2 and Si:HfO2-based MFM capacitors. *Applied Physics Letters* **2021,** *118* (6).

(12) Johansson, S.; Memisevic, E.; Wernersson, L. E.; Lind, E., High-Frequency Gate-All-Around Vertical InAs Nanowire MOSFETs on Si Substrates. *IEEE Electron Device Letters* **2014,** *35* (5), 518-520.

(13) Mulaosmanovic, H.; Breyer, E. T.; Mikolajick, T.; Slesazeck, S., Reconfigurable frequency multiplication with a ferroelectric transistor. *Nature Electronics* **2020,** *3* (7), 391-397.
